# Mutagenic effects of nitrogen protoxide and oryzalin on “41 B” and “Fercal” grapevine rootstocks seedlings

**DOI:** 10.1270/jsbbs.23003

**Published:** 2023-09-09

**Authors:** Zeki Kara, Osman Doğan

**Affiliations:** 1 Department of Horticulture, Faculty of Agriculture, Selcuk University, Konya, 42250, Turkey

**Keywords:** breeding, chromosome count, grape rootstock, flow cytometry, mutagen, polyploidy

## Abstract

In this study, the mutagenic effects of different doses and exposure times of oryzalin and Nitrogen Protoxide (N_2_O) were tested for stimulating polyploid on 41 B and Fercal grapevine rootstocks seedlings. Ploidy changes were examined by morphological, cytological, macroscopic, and microscopic methods. Leaf thickness, chlorophyll contents, stomatal sizes, and chloroplast numbers of polyploid seedlings stimulated with mutagens increased but their stomatal densities decreased. Flow cytometry (FC) analyses were performed on 50 samples selected by morphological and microscopic preliminary determinations. In FC analyses, 1 tetraploid seedling and 4 mixoploid seedlings from Fercal offspring and 1 mixoploid seedling from 41 B offspring were verified. The nuclear DNA content of tetraploid and mixoploid seedlings were increased by 2.00 and 1.34-fold, respectively, when compared to their diploid parents. Chromosome counts in root tip samples propagated *in vitro* from the tetraploid Fercal offspring confirmed a 2-fold increase compared to the diploid parent. In polyploidy induction studies, it was deemed appropriate to use FC analysis and chromosome count together to confirm the ploidy levels of mutants. Oryzalin and N_2_O applications at different doses and exposure times were found to be effective for inducing polyploidy in 41 B and Fercal grapevine rootstocks.

## Introduction

Due to its socio-economic value, viticulture is carried out in various regions and climates all over the world, and therefore, diversity is needed in both grape and rootstock varieties. Over 25000 grape varieties and 1435 grape rootstocks have been registered in the world (VIVC 2023). However, about 10 rootstock varieties are used in 90% of the vineyards globally (Keller 2020), and approximately 50% of them are rootstocks obtained by Teleki/Kober selection (Reynolds 2015). It is practically not possible for the few rootstock varieties currently used to meet all the demands of these different vineyard areas (Reynolds 2015).

Following the destructive effect of phylloxera and the necessity to use rootstocks, rootstock breeding was started in France and 862 vine rootstocks were identified until today, and 171 vine rootstocks were identified in Italy, which started later. Grapevine rootstock breeding is also studied in countries such as Germany, Hungary, and Czech Republic. Although the vineyards are mostly established on their own roots in China, which has the largest vineyard area today, rootstock breeding has intensified in the last 10 years (Reynolds 2015, VIVC 2023). Also, rootstock breeding has been studied in Korea for the last 20 years (Yun and Park 2007).

Due to the heterozygous nature of the grapevine, more than 274 billion different possible combinations of genotypes are formed when hybridized. Therefore, the prediction of hybridization results is difficult, and it is extremely low to reach defined chromosome combinations by crossing (Reynolds 2015).

Polyploidy is the presence of more than two genomes per somatic cell in the organism. Polyploid organisms, it may occur spontaneous with chromosome copies of somatic cells (mitotic ploidy) or in meiosis due to the failure to separate homologous chromosomes that cause diploid gametes (Ramsey and Schemske 2002). Polyploidy can also be induced artificially in the laboratory by applications of mutagen that inhibit cell division. Polyploids are known to provide some selective advantages compared to diploids. Some of these are differentiating growth power, increased heterozygosity, new variations, obtaining new genotypes and allelic sub-functionalization (Abbott *et al.* 2007, Anssour *et al.* 2009, Dar *et al.* 2013, Gao-Takai *et al.* 2017, Motosugi *et al.* 2007, Salmon *et al.* 2005, Udall and Wendel 2006).

With the increased interest in the tetraploid grape varieties, polyploidy studies intensively are conducted in China (Chen *et al.* 2014), South Korea (Park *et al.* 2006), Japan (Yamada and Sato 2016), Brazil (Sinski *et al.* 2014) and Turkey (Kara *et al.* 2018b). In addition, the development of tetraploid rootstocks to solve the problems posed by grafting tetraploid grape varieties to diploid rootstocks is a relatively new area of interest. Dwarf tetraploid grapevine rootstocks provide a decrease in vigor, so they can be used as dwarf rootstock. Thus, it is possible to establish vineyards in high density (Gao-Takai *et al.* 2017). In addition, it was reported that by grafting tetraploid grape varieties onto tetraploid rootstocks, the coloration of the fruits (Gao-Takai *et al.* 2017, Motosugi *et al.* 2007) and the anthocyanin content (Gao-Takai *et al.* 2017) increased compared to those on diploid rootstocks.

Colchicine is the most used mutagen in polyploidy studies (Kosonoy-González *et al.* 2019). Successful polyploidy promotion requires a synergistic match of the effective penetration of the antimitotic agent and may depend on exposure time and dose of antimitotic agents, tissue types, and interactions with basal media and plant growth regulators (Touchell *et al.* 2020). When the right application time and dose is selected, N_2_O can easily penetrate the plant and promote polyploidy due to its small molecule structure. In addition, N_2_O is not as harmful to nature as colchicine (Kara *et al.* 2018c). Oryzalin, on the other hand, is an herbicide that depolymerizes the mitotic spindle at appropriate concentrations with the same mechanism of action as colchicine, stimulates the formation of a greater amount of polyploid cells and provides a higher polyploidy efficiency (Kosonoy-González *et al.* 2019).

Polyploidy is a popular field in plant breeding that has been extensively studied in the last century. Obtaining polyploid plants from numerous species has increased the interest in studies of inducing polyploidy with mutagens and different protocols have been applied by researchers (Touchell *et al.* 2020). Today, many types of polyploid varieties are cultivated in some countries. However, the number of polyploid rootstocks suitable for these varieties is almost negligible. The gap of this area reveals the necessity of polyploid rootstock breeding.

In this study, the effects of N_2_O on stratified seeds of 41B and Fercal rootstocks, and oryzalin applications on polyploid plant induction during the development of cotyledon leaves in seedlings were examined macroscopically, microscopically, and cytologically. Rapid cell divisions take place at the stage when seeds begin to germinate, and cotyledon leaves are formed. Mutagens are thought to be more effective in rapid cell division. Therefore, N_2_O was applied to germinated seeds and oryzalin was applied at the stage of cotyledon leaves formation.

## Materials and Methods

The seeds of 41 B [Chasselas (*Vitis vinifera* L.) × (*Vitis berlandieri* Planch.)] and Fercal [(*Vitis vinifera* L. × *Vitis berlandieri*) × 333 EM (Cabernet-Sauvignon × *Vitis berlandieri*)] grapevine rootstocks in a moist perlite at +4°C for 90 days were used as a plant material. With the onset of the first germination, N_2_O applied seeds at various doses (2.5, 5 and 10 bar) and exposure times (48-h and 96-h) were planted in germination pans. In addition, seeds that were not treated with N_2_O were sown in plastic viol and 25 and 100 μM oryzalin were applied with a Pasteur pipette for 2 days (48 h) and 4 days (96 h) twice a day (08:30 and 18:00) during the formation of cotyledon leaves. Control plants were not mutagenized during the seed germination period in which N_2_O was applied and the cotyledon leaves developed when oryzalin was applied. Only pure water was dripped onto the growth zones with a Pasteur pipette for 4 days (96 h). Mutagen-treated plant materials were planted in peat:perlite (3:1) medium in the greenhouse. N_2_O and oryzalin mutagen agents were applied to a total of 1200 plants in the same amount for each of 41 B and Fercal rootstocks. The effects of mutagens were investigated by the survival rates of plants, stomatal density (number mm^–2^), stoma size (μm), chloroplast numbers in stoma guard cells (number stoma^–1^), leaf thickness and chlorophyll amount (SPAD value) in developing plants. In addition, FC analysis, nuclear DNA contents and chromosome counting were determined within the scope of cytological examinations.

### Ploidy analysis and determination of nuclear DNA content by flow cytometry (FC)

Initially, 50 seedlings thought to be polyploid based on chloroplast count results were selected. Ploidy confirmation test was performed by FC analysis on fresh leaf samples of these selected seedlings. For FC analysis, fresh leaf samples were taken in 0.5 cm^2^ pieces, placed in a petri dish, 500 μL isolation buffer (Partec-Nuclei Extraction Buffer) was added and the leaf tissue was chopped with a razor blade until it was cut into small pieces. Thus, the cell nuclei were released, and openings were created on the nuclear membrane. The samples in the Petri dish were shaken for 10–15 seconds, filtered with Partec-CellTrics 30 μm-green filter, and transferred to tubes (Partec-Sample Tubes, 3.5 mL, 55 × 12 mm). 1600 μL of staining solution [Partec-DAPI (4,6 diamidino-2-phenylindole) Staining Buffer] was added and left in an environment with light isolation for 5 minutes. Subsequently, samples were analyzed in FC (Kara and Yazar 2022). The nuclear DNA contents of the grapevine samples were compared with tomato whose DNA content is known.

Calculation of nuclear DNA content was done by the following formula (Cimen 2020, Seker *et al.* 2003):

$$\text{Sample }2\text{C value }\left(\text{DNApg}\right)=\frac{\text{Sample mean peak position}\times \text{Reference }2\text{C value}}{\text{Reference }2\text{C mean peak position}}$$

### Chromosome counting

As an additional confirmation procedure, chromosomes were counted in root tip cells in plants that survived after mutagen applications and whose ploidy levels were confirmed by FC analyses. Selected polyploid rootstock seedlings were propagated *in vitro* MS medium (Kara *et al.* 2022, Murashige and Skoog 1962). Root tip samples were taken early in the morning (10:30–11:00) for chromosome counting. As a pre-treatment, the samples were kept in 0.002 M 8-hydroxyquinoline solution at +4°C for 8 hours. Then it was washed with distilled water and fixed in Carnoy solution for 8 hours at +4°C. Hydrolysis was carried out by keeping it in 5 N HCl acid for 30 minutes at room temperature. Then, the root tips were washed with pure water. At the staining stage, the root tissues were stained by keeping them in 2% aceto-orcein at +4°C for 1–2 days (Kara and Yazar 2022, Uysal *et al.* 2009). Chromosomes were counted from cells at the appropriate metaphase stage and their pictures were recorded with a Zeiss Axio microscope-Zeiss Axiocam 105 Color digital camera.

### Data analysis

The data obtained because of oryzalin and N_2_O treated 41 B and Fercal rootstocks were compared in SPSS 17.0 statistical program (SPSS Inc, Chicago, IL, USA) Duncan multiple comparison test, dose and duration applications JMP 7 statistical programs with Student’s t test at p < 0.05 significance level.

## Results

### Viability rate (%) and LD_50_ value

**Fig. 1.**
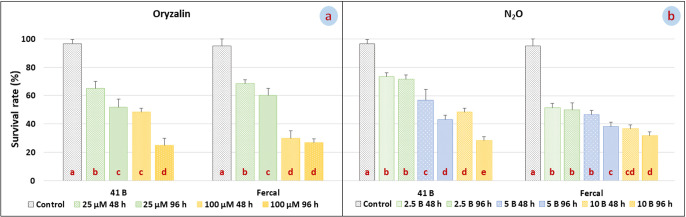
Effects of oryzalin (a) and N_2_O (b) applications on viability rates. LSD values of 41 B-oryzalin, Fercal-oryzalin, 41 B-N_2_O and Fercal-N_2_O applications, respectively: % 8.85, %7.97, %7.52 and %6.95.

The effects of mutagen applications on viability rates were significant. The viability rates of both oryzalin and N_2_O treated plants were lower than the control. The lowest viability rate in oryzalin applications was determined at 100 μM- 96-h application to 41 B grapevine rootstock (Fig. 1a). In N_2_O application, the lowest viability rate was at 10 bar- 96-h application to 41 B grapevine rootstock (Fig. 1b).

The LD_50_ values of the grapevine rootstocks treated with oryzalin and N_2_O were estimated by linear regression based on the lethal rate (Table 1). The LD_50_ values of Oryzalin applications for 48-h and 96-h were determined as 87.07 and 27.26 μM in 41 B rootstock, 48.51 and 37.89 μM in Fercal, respectively. LD_50_ values obtained by N_2_O applications were determined as 8.44 and 4.66 bar for 41 B, 3.15 and 2.35 bar for Fercal, respectively. Rootstock genotype was important in LD_50_ values. The LD_50_ values of both mutagen applications were higher in 41 B than that of Fercal rootstocks.

### Polyploid plants

**Fig. 2.**
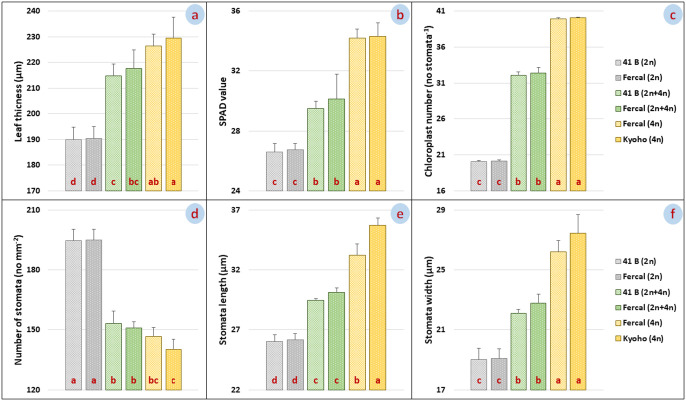
Leaf thickness (a), SPAD value (b), chloroplast number (c), stomata number (d), stomatal length (e) and stomatal width (f) of diploid, myxoploid and tetraploid plants. LSD values of eaf thickness (a), SPAD value (b), chloroplast number (c), stomata number (d), stomatal length (e) and stomatal width (f), respectively: 11.01 μm, 1.61 mg kg^–1^, 0.67 no stomata^–1^, 8.50 no mm^–1^, 1.08 μm and 1.49 μm.

Differences between leaf thickness, SPAD values, chloroplast numbers, stomata numbers, stomatal length and width data of selected seedlings and control seedlings [41 B (2n), Fercal (2n), 41 B (2n + 4n), Fercal (2n + 4n), Fercal (4n) and Kyoho (4n)], whose ploidy level was detected by FC analysis with our mutagen stimulation, were significant. Leaf thicknesses of selected seedlings and control plants were measured as 190.05 ± 4.78 μm, 190.44 ± 4.54 μm, 214.68 ± 4.76 μm, 217.68 ± 7.27 μm, 226.38 ± 4.65 μm and 229.50 ± 8.08 for 41 B (2n), Fercal (2n), 41 B (2n + 4n), Fercal (2n + 4n), Fercal (4n) and Kyoho (4n), respectively (Fig. 2a). The leaf thicknesses of the selected polyploid seedlings were significantly greater than the diploid control parent leaves. SPAD data were determined as 26.61 ± 0.56, 26.74 ± 0.42, 29.48 ± 0.50, 30.14 ± 1.63, 34.20 ± 0.60 34.31 ± 0.91 SPAD values for 41 B (2n), Fercal (2n), 41 B (2n + 4n), Fercal (2n + 4n), Fercal (4n) and Kyoho (4n), respectively (Fig. 2b). Parallel to the increase in ploidy level, an increase was observed in SPAD values. The highest SPAD value was recorded in cv. Kyoho, while the closest value was in the selected tetraploid Fercal offspring.

Chloroplast numbers in stoma guard cells were determined as 20.09 ± 0.18, 20.17 ± 0.15, 32.07 ± 0.49, 32.40 ± 0.78, 39.93 ± 0.14, 40.03 ± 0.12 stoma^–1^ for 41 B (2n), Fercal (2n), 41 B (2n + 4n), Fercal (2n + 4n), Fercal (4n) and Kyoho (4n), respectively (Fig. 2c). The chloroplast numbers of mixoploid plants were approximately 1.5-fold higher than diploids, while it was approximately 2-fold higher in tetraploid plants.

The number of stomata per unit area (mm^2^) were determined as 194.58 ± 5.92, 194.94 ± 5.40, 153.27 ± 6.13, 150.94 ± 3.24, 146.66 ± 4.68 and 140.24 ± 4.99 for 41 B (2n), Fercal (2n), 41 B (2n + 4n), Fercal (2n + 4n), Fercal (4n) and Kyoho (4n), respectively (Fig. 2d). The lowest stomatal density was in the cv. Kyoho, while the highest stomatal density was in the diploid Fercal control plants. As the ploidy level increased, a decrease in stomatal density was noted. Stomatal length data of selected offsprings and control plants were determined as 26.03 ± 0.59 μm, 26.19 ± 0.49 μm, 29.45 ± 0.18 μm, 30.12 ± 0.40 μm, 33.25 ± 0.94 μm and 35.72 ± 0.60 μm for 41 B (2n), Fercal (2n), 41 B (2n + 4n), Fercal (2n + 4n), Fercal (4n) and Kyoho (4n), respectively (Fig. 2e), while stomatal widths were 19.01 ± 0.75 μm, 19.08 ± 0.66 μm, 22.12 ± 0.25 μm, 22.79 ± 0.61 μm, 26.20 ± 0.78 and 27.45 ± 1.25 μm in the same order (Fig. 2f). The lowest stomatal length and width were measured in the diploid 41 B control plants, while the highest values were recorded in the cv. Kyoho. The increase in ploidy level also increased the stomatal length and width.

### FC analysis and nuclear DNA contents

**Fig. 3.**
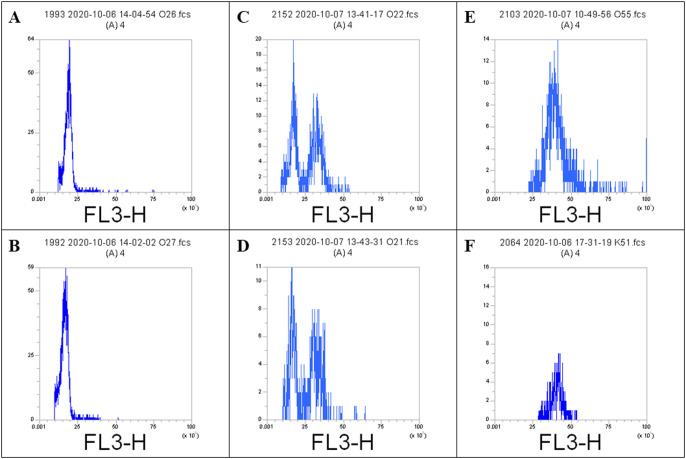
FC histograms of nuclei isolated from 2n Fercal (A), 2n 41 B (B), 2n + 4n Fercal (C), 2n + 4n 41 B (D), 4n Fercal (E) and 4n Kyoho (F) grapevine rootstock leaves.

As a result of chloroplast count, a total of 50 seedlings from all applications had polyploidy preliminary data and FC analysis was performed to verify the ploidy level of these samples. As a result of FC analysis, 5 mixoploid (100 μM-96-h) and 1 tetraploid (10 bar-96-h) seedling were detected. Four of the mixoploid plants were Fercal and one was the 41 B offspring. Only one Fercal offspring was confirmed to be tetraploid (Fig. 3). In FC analysis, the ploidy level is determined by its correlation with the relative or absolute DNA content-DNA ploidy level. Therefore, if an increase in DNA content corresponds to increases in chromosome number, the DNA content of a sample with a known ploidy level can be used as a reference standard to determine the DNA ploidy level of an unknown sample (Doležel *et al.* 2007).

The nuclear DNA content of rootstock offspring’s, which was confirmed by FC analysis, was significantly different. Nuclear DNA contents of 41 B (2n), Fercal (2n), 41 B (2n + 4n), Fercal (2n + 4n), Fercal (4n) and Kyoho (4n) plants were at the levels of 1.034, 1.115, 1.477 (0.99 + 1.97), 1.510 (1.02 + 2.00), 2.110 and 2.144 pg, respectively (Table 2). While the nuclear DNA content of the tetraploid plant was determined to be twice as compared to diploids, it was found to be approximately 1.5-fold in mixoploid plants.

### Chromosome counting

**Fig. 4.**
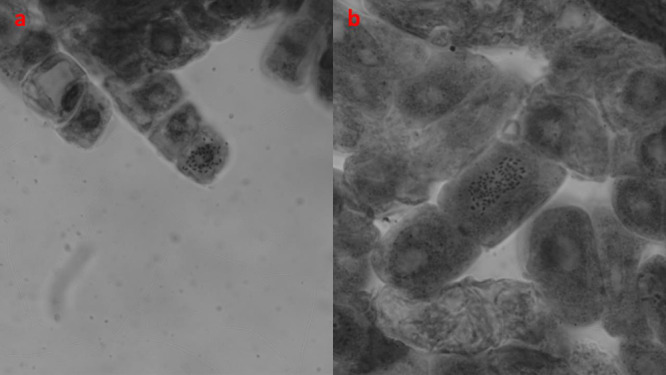
Chromosome counts of the control 2n Fercal (a: 2n = 38) and the 4n Fercal (b: 2n = 4x = 76) offspring.

Chromosomes of the tetraploid Fercal (selected from 10 bar 96-h N_2_O application) offspring, whose increase in ploidy level was confirmed by FC analysis, were counted in preparations prepared by root tip crushing technique. The Fercal offspring, which was determined to be polyploid (tetraploid) by FC analysis, was propagated by *in vitro* nodal culture, and chromosomes were counted in fresh root tip samples. In addition, diploid Fercal grapevine rootstock was also propagated same method, and chromosomes were counted for control purposes. The chromosome number of diploid Fercal was 2n = 38 (Fig. 4a), and the chromosome number of the tetraploid Fercal offspring was 2n = 4x = 76 (Fig. 4b). Root tip chromosome counts confirmed the FC analysis. Double validation was performed on the selected polyploid Fercal offspring.

## Discussion

In this study, all Oryzalin and N_2_O applications to germinated seeds and seedlings of 41 B and Fercal rootstocks decreased the viability rates. In previous similar studies, oryzalin (Xie *et al.* 2015, Zakizadeh *et al.* 2020, Zeng *et al.* 2019) and N_2_O (Molenaar *et al.* 2018) applications decreased the viability rate. In some studies, it has been reported that oryzalin is highly toxic to plants, especially at high concentrations and long exposure times (Dhooghe *et al.* 2009, Dunn and Lindstrom 2007). In general, low doses and short exposure times increased the survival rate while decreasing the acquisition frequency of tetraploid plants (Chakraborti *et al.* 1998, Väinölä 2000, Zhang *et al.* 2008). In polyploidy studies, it was suggested that mutagen applications inducing polyploidy would not be a disadvantage, reducing the viability rate (Väinölä 2000).

The median lethal dose (LD_50_) has often been used as a critical parameter for chemical mutagenic agents (Chen *et al.* 2020). In previous studies, LD_50_ values were calculated in two ways. The first method, a 50% mortality rate were given as the LD_50_ value (Asoko *et al.* 2020, Cabahug *et al.* 2020). In the second method, lethal dose-based linear regression was used (Chen *et al.* 2020, Pal *et al.* 2017). In our study, time- and dose-dependent LD_50_ value was determined by dose-based linear regression. In our study, LD_50_ values decreased in contrast to mutagen application time or dose increase, these results supported previous studies (Chen *et al.* 2020, Hasim *et al.* 2021, Kerdsuwan and Te-chato 2012, Mahajan *et al.* 2015, Pal *et al.* 2017).

The leaves of polyploid plants comparison on diploid originals are thicker (Rameshsing *et al.* 2015, Zhou *et al.* 2017), darker green (Chen *et al.* 2011, He *et al.* 2012), wider (Ishfaq *et al.* 2012, Zhou *et al.* 2017), toothed and rounded edges (Tang *et al.* 2010), larger (Dudits *et al.* 2016, Xu *et al.* 2016), abnormal spata (bud-leaf) and pedicel (Chen *et al.* 2011) have varying shapes and structures.

It was also reported that leaf thickness of tetraploid plants obtained with oryzalin applications increased compared to their diploid origin (Lu *et al.* 2014, Zeng *et al.* 2019). Similarly, Bae *et al.* (2020) reported that the leaves of tetraploid plants show a small, thick, and wrinkled structure compared to their diploids. The leaf thicknesses of our stimulated plants confirm previous studies.

In addition, in previous polyploid studies, it was reported that the chlorophyll content of tetraploid plants increased compared to diploids (Mo *et al.* 2020, Rao *et al.* 2019). In contrast, Atichart and Bunnag (2007) determined that the leaf chlorophyll content of the polyploid *Dendrobium secundum* (Bl.) Lindl was significantly less than the original diploids.

Fu *et al.* (2019) determined that the chlorophyll content of tetraploid herbs was 1.76-fold higher than diploids. In another study, they determined that the chlorophyll content in the leaves of tetraploid plants was 1.40-fold higher than that of diploids (Li *et al.* 2021). Our data on chlorophyll content in induced plants are similar the literature reporting to increase.

The chloroplast numbers in stoma guard cells are an effective method to determine the ploidy level (Zhang *et al.* 2005). It was reported that the chloroplast numbers of tetraploid plants were twice that of diploids (Rao *et al.* 2019, Rêgo *et al.* 2011). It was also known that the number of chloroplasts in stoma guard cells is associated with ploidy (Sinski *et al.* 2014, Zhang *et al.* 2010). The chloroplast number of our polyploid plants increased our results were compatible with the literature (Rao *et al.* 2019, Rêgo *et al.* 2011).

Stoma sizes were used as an indirect method for the identification of polyploids (Moghbel *et al.* 2015). In general, stomatal properties were used for the rapid and early identification of polyploids. In addition, the method used to detect stomatal properties is simple and does not require expensive instruments (Cohen and Yao 1996, Gu *et al.* 2005, Tang *et al.* 2010). All treatments on two grapevine rootstocks caused a decrease in the number of stomata and an increase in the size of the stoma compared to the control. Kara *et al.* (2018c) found that N_2_O applications to 41 B grapevine rootstocks reduced stomatal density by 28%. Bae *et al.* (2020) also identified a similar decrease in stomatal density in oryzalin applications. The results obtained with oryzalin and N_2_O applications in previous studies were also supported by our findings.

Stoma size increase has often been used to describe chromosome doubling (Dhooghe *et al.* 2011, Stanys *et al.* 2006). In a similar previous study, Omezzine *et al.* (2012) reported that the stomata length of mixoploid plants was significantly greater than that of diploids. Zeng *et al.* (2019) also found a significant increase in stomatal sizes of polyploid plants formed because of the oryzalin application compared to their originals. Also, stomatal width results were like stomatal length data. It is known that the stomatal widths of diploid, mixoploid and tetraploid plants differ significantly (Cimen 2020). In previous studies, it was found that the applications of oryzalin increased the width of the stomata (Bae *et al.* 2020, Fakhrzad *et al.* 2023, Pliankong *et al.* 2017).

One of the most notable advanced agronomic features of tetraploid plants is the change in stomatal size (Kosonoy-González *et al.* 2019). The stomatal sizes of polyploid plants are larger than diploid plants and the stomatal density per unit is lower (Bae *et al.* 2020, Kara *et al.* 2018c, Lu *et al.* 2014, Yang *et al.* 2006). This difference is probably due to an increase in cell size in polyploid individuals (Marinho *et al.* 2014) and/or a decrease in leaf mesophyll porosities (Lundgren *et al.* 2019). The stomatal characteristics (Bae *et al.* 2020, Kara *et al.* 2018a, Zeng *et al.* 2019) and chloroplast numbers (Ewald *et al.* 2009, Rao *et al.* 2019, Rêgo *et al.* 2011) of polyploid plants obtained in this study were like previous studies, and our results confirmed previous studies.

FC analysis is a fast, reliable, and simple method for determining the ploidy level and verifying the success of polyploidy induction and enables analysis of many target plants in a short time (Roy *et al.* 2001). Also, FC is the foremost method for evaluating induced polyploidization (Dhooghe *et al.* 2011). FC is a fast and precise method used to determine the nuclear DNA content of plants (Galbraith *et al.* 1983). It can also be used efficiently for ploidy determination in the field, greenhouse, and *in vitro* grown plants (Joachimiak *et al.* 2001, Sliwinska and Steen 1995, Thiem and Śliwińska 2003). This technique is more accurate than traditional methods such as stoma measurements and faster than chromosome counts (O’Brien *et al.* 1996, Ollitrault-Sammarcelli *et al.* 1994, Sgorbati *et al.* 1986, Thao *et al.* 2003, Tosca *et al.* 1995, Väinölä 2000).

The nuclear DNA content of the Vitaceae family was between 0.62 and 1.24 pg (Chu *et al.* 2018), nine different diploid *Vitis vinifera* varieties ranged from 1.17 to 1.26 (Leal *et al.* 2006), diploid Tekirdag Muscat and Michele Palieri varieties were 1.10 and 1.20 (Ergönül *et al.* 2018), and diploid 41 B grapevine rootstocks were reported as 1.028 pg (Kara *et al.* 2018a). Gordej *et al.* (2019) reported that the nuclear DNA content of tetraploid plants obtained because of N_2_O application increased 1.7 to 1.82-fold compared to diploids. Another similar study (Cimen 2020) found that it increased 2.01-fold. The nuclear DNA content results in our study supports previous studies.

Chromosome counting is the most concrete method to determine the ploidy level in polyploidy studies (Dhooghe *et al.* 2011, Doležel *et al.* 2007). However, this method is very laborious and only a limited number of cells can be analyzed (Bohanec 2003, Dhooghe *et al.* 2011, Xie *et al.* 2015).

In previous studies, chromosome numbers (Doležel *et al.* 2007, Vilcherrez-Atoche *et al.* 2023), FC analysis (Doležel *et al.* 2007, Eeckhaut *et al.* 2005) and stomatal parameters (Cohen and Yao 1996, Tal 1980) were used to determine the ploidy level of plants. Xie *et al.* (2015) reported that both chromosome count and FC analyses are accurate methods to determine the ploidy level and there is a significant correlation between the results obtained from the two methods. In polyploidy induction studies, the most reliable methods for confirming the ploidy levels of mutated plants is FC analysis and chromosome count, and it is thought that using these two methods together is more beneficial than using them alone.

The applications in the study made had a significant effect on the survival rate compared to the control. Although stomatal characteristics, an indirect method for detecting polyploidy, did not provide definitive results, it did provide the first screening opportunity to estimate the level of polyploidy when working with many (in this study 1200 mutagen induced) plants. Significant increases were determined in leaf thickness stomatal sizes, chloroplast numbers, and chlorophyll content but decrease stomatal densities of polyploid individuals, which induced by oryzalin and N_2_O. One tetraploid and five mixoploid plants were detected by FC analysis of 50 samples we selected preliminary data. The tetraploid plant was obtained by the N_2_O application, while mixoploid plants were induced by oryzalin applications. The nuclear DNA content of mixoploid and tetraploid individuals was increased compared to diploid plants. Our study proved for the first time that N_2_O gas can be used successfully to obtain a polyploid individual in grapevine rootstocks. The stomatal characteristics and chloroplast numbers, which were used as preliminary data in confirming the polyploidy of the applications and detecting the polyploid individuals, were partially successful. FC analysis was indispensable in detecting the mixoploid genotypes. FC analysis for the detection of ploidy levels of mutants obtained in polyploidy induction and chromosome count were deemed necessary for definitive confirmation. Similar doses and exposure times of oryzalin and N_2_O can be used for different genotypes in the next studies to induce polyploidy with mutagens.

## Author Contribution Statement

ZK and OD planned to research. OD carried out seed germination and mutagen applications. ZK and OD analyzed morphological features. OD performed FC analysis and chromosome count. ZK and OD did statistical analysis and wrote the article. ZK supervised OD’s PhD study.

## Figures and Tables

**Table 1.** LD_50_ values of oryzalin and N_2_O applications

Rootstock	41 B		Fercal	
Duration	48 hours	96 hours	48 hours	96 hours
Oryzalin (μM)	87.07	27.26	48.51	37.89
N_2_O (Bar)	8.44	4.66	3.15	2.35

**Table 2.** Nuclear DNA content of diploid and polyploid plants

Ploidy level	41 B (2n)	Fercal (2n)	41 B (2n + 4n)	Fercal (2n + 4n)	Fercal (4n)	Kyoho (4n)	LSD_0.05_
Genome size (pg 2C^–1^)	1.034^c^	1.115^c^	1.477 (0.99 + 1.97)^b^	1.510 (1.02 + 2.00)^b^	2.110^a^	2.144^a^	0.128
Standard deviation	0.041	0.098	0.056 (0.05 + 0.07)	0.110 (0.10 + 0.12)	0.024	0.015	
